# High-Fidelity Simulators in Undergraduate Medical Education: A Systematic Review

**DOI:** 10.7759/cureus.95019

**Published:** 2025-10-20

**Authors:** Urshila Ramah, Bibi Sumera Keenoo

**Affiliations:** 1 Public Health, University of Mauritius, Reduit, MUS

**Keywords:** high fidelity simulation training, medical student teaching, simulation in medical education, skill training, undergraduate teaching

## Abstract

High-fidelity simulators (HFS) have become integral to undergraduate medical education (UME), offering realistic clinical training and skill development opportunities. Despite their growing use, the comparative effectiveness of HFS versus low-fidelity simulators (LFS) and traditional teaching methods remains unclear. This review evaluates the impact of HFS in UME, focusing on skill acquisition, knowledge retention, student confidence, and cost-effectiveness.

A systematic review was conducted following Preferred Reporting Items for Systematic Reviews and Meta-Analyses (PRISMA) guidelines. We searched PUBMED, Ovid, and LibSearch for studies from 2000 to 2025 comparing HFS with LFS or traditional teaching methods in UME. Forty-four studies met the inclusion criteria and were critically appraised.

HFS significantly improved procedural skills, particularly in high-stakes scenarios such as emergency medicine. They also enhanced student confidence in complex clinical situations. However, evidence on the impact of HFS on knowledge retention was mixed, and LFS were found to offer similar outcomes for basic skills training at a lower cost.

HFS play a valuable role in UME, especially for complex skills, but a tiered approach utilizing LFS for basic training may offer cost-effective alternatives. Future research should evaluate the long-term effects of HFS on clinical practice.

## Introduction and background

Medical education has undergone a significant transformation in recent decades, shifting from traditional didactic lectures toward more practical and immersive learning approaches. High-fidelity simulators (HFS) have become an integral component of undergraduate medical education (UME), offering realistic clinical scenarios in which students can practice both technical and non-technical skills in a safe, controlled environment [1]. By replicating real patient care situations, HFS enable learners to bridge theoretical knowledge with clinical practice, while fostering decision-making, teamwork, and communication skills without compromising patient safety [2].

The growing adoption of simulation-based education is driven by the dual need to enhance patient safety and to better prepare students for complex clinical environments. However, debate remains regarding the comparative value of HFS versus low-fidelity simulators (LFS) and traditional teaching methods, particularly in relation to skill acquisition, knowledge retention, learner confidence, and cost-effectiveness [3]. While HFS provide highly immersive experiences, evidence suggests that LFS may achieve comparable outcomes for certain basic skills at a fraction of the cost [4].

This systematic review aims to evaluate the educational impact of HFS in UME, with a particular focus on skill acquisition, knowledge retention, confidence building, and cost-effectiveness. Furthermore, it compares HFS with LFS and traditional instructional methods, assessing the strength of available evidence to inform best practices in medical education.

## Review

Methodology

Search Strategy

This systematic review was conducted in accordance with the Preferred Reporting Items for Systematic Reviews and Meta-Analyses (PRISMA) guidelines [3]. A comprehensive search was carried out in PubMed, Ovid, and LibSearch to identify relevant studies published between 2000 and 2025. Search terms included “high-fidelity simulators,” “low-fidelity simulators,” and “undergraduate medical education.”

Eligibility Criteria

**Figure 1:**
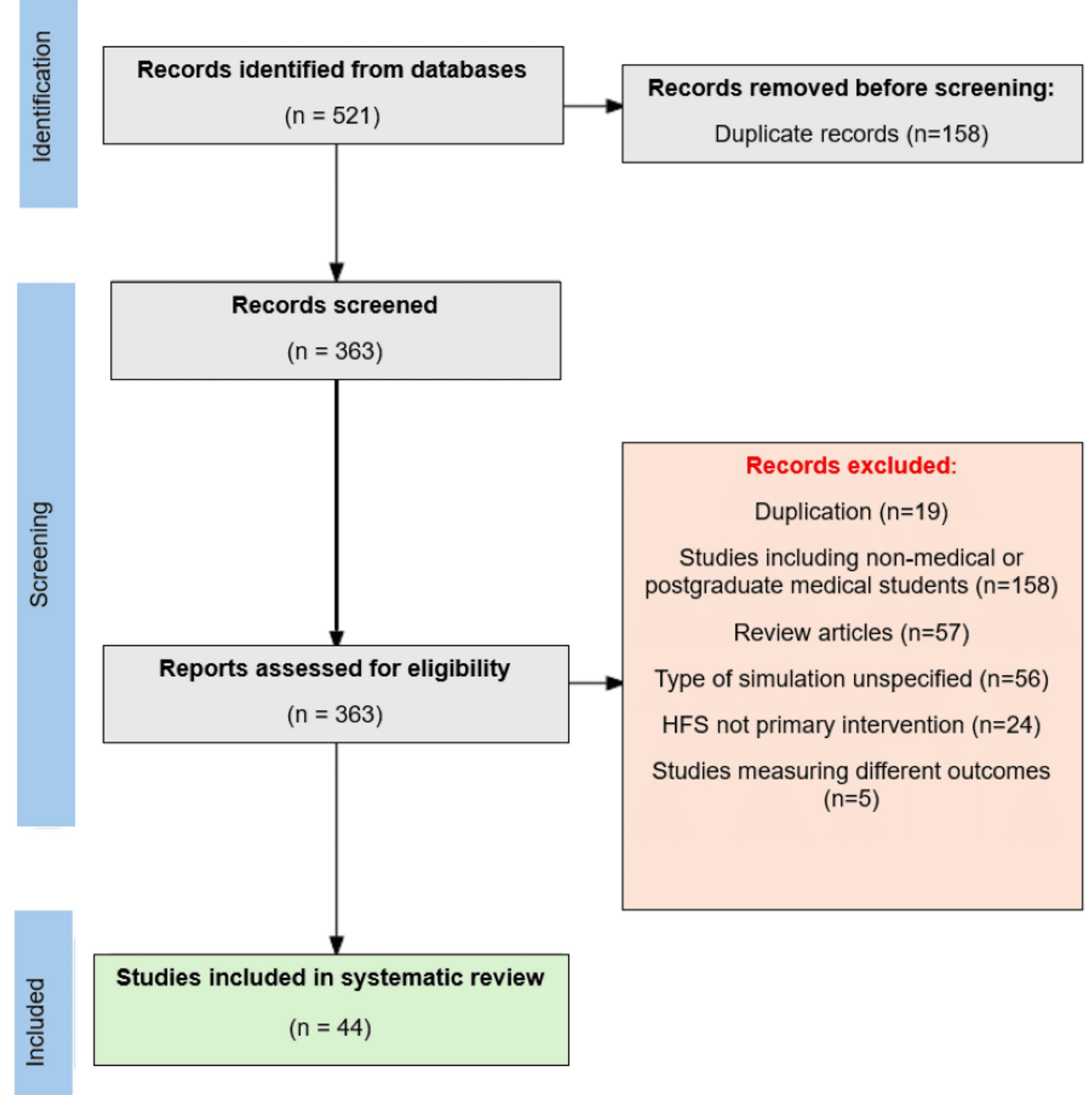
PRISMA flow diagram showing the identification, screening, and inclusion of studies in the systematic review. PRISMA: Preferred Reporting Items for Systematic Reviews and Meta-Analyses; HFS: high-fidelity simulators.

Studies were eligible for inclusion if they focused on undergraduate medical students, utilized HFS as the primary intervention, and compared HFS with either LFS or other teaching methods. Eligible studies were randomized controlled trials (RCTs), prospective studies, and retrospective studies published in English. Studies not explicitly focusing on UME were excluded. The PRISMA flow diagram (Figure 1) illustrates the process of study identification, screening, eligibility assessment, and final inclusion.

Data Extraction and Analysis

The studies were screened by two reviewers independently. Data were extracted using a pre-designed standardized form, capturing key details including study design, sample size, type of simulation, measured outcomes (such as skill acquisition, knowledge retention, student confidence, and cost-effectiveness), and other relevant findings. Any discrepancies in data extraction were resolved through consensus between the two reviewers. To assess risk of bias, we applied the Cochrane Risk of Bias Tool (RoB 2) for RCTs and the Risk Of Bias In Non-randomized Studies of Interventions (ROBINS-I) for non-randomized studies.

Results

Study Characteristics

A total of 44 studies were included in this review (Table 1). Twenty studies were RCTs, 22 were prospective studies, and two were retrospective studies. Most studies focused on clinical specialties such as emergency medicine, obstetrics, surgery, and internal medicine. Sample sizes ranged from 12 to 200 participants, and the studies varied in terms of their design and outcomes measured. Outcomes included skill acquisition, knowledge retention, and student confidence. Traditional teaching methods included didactic lectures and case-based discussions.

**Table 1:** Summary of the studies included in the systematic review. HFS: high-fidelity simulators; LFS: low-fidelity simulators; RCT: randomized controlled trial.

Study	Design	Participants	Intervention	Outcomes measured	Setting/place
Barsuk et al., 2009 [5]	RCT	100	HFS vs. LFS	Skill acquisition	United States
Conlon et al., 2014 [6]	Prospective	80	HFS vs. LFS	Knowledge retention	Canada
Norman et al., 2012 [7]	RCT	120	HFS vs. traditional	Skill acquisition	United Kingdom
Yang et al., 2019 [8]	Prospective	60	HFS	Student confidence	China
Goolsby et al., 2014 [9]	Prospective	90	HFS vs. LFS	Knowledge retention	United States
Massoth et al., 2019 [10]	RCT	110	HFS	Skill acquisition	Germany
Lefor et al., 2020 [4]	Prospective	70	HFS vs. LFS	Student confidence	Japan
McCoy et al., 2019 [11]	RCT	50	HFS vs. traditional	Skill acquisition	United States
Kodikara et al., 2019 [12]	Prospective	200	HFS vs. LFS	Knowledge retention	Sri Lanka
Feinstein et al., 2001 [13]	RCT	75	HFS	Skill acquisition	United States
Armenia et al., 2018 [14]	RCT	65	HFS vs. LFS	Student confidence	United Kingdom
Adler et al., 2007 [15]	Prospective	55	HFS	Skill acquisition	United States
Price et al., 2010 [16]	RCT	130	HFS vs. traditional	Knowledge retention	Canada
Arcoraci et al., 2019 [17]	RCT	100	HFS vs. LFS	Skill acquisition	Italy
Holling et al., 2015 [18]	Prospective	45	HFS	Knowledge retention	United Kingdom
Nicolaides et al., 2020 [19]	RCT	85	HFS vs. traditional	Student confidence	Greece
Swamy et al., 2014 [20]	RCT	150	HFS vs. LFS	Skill acquisition	United Kingdom
Cavuoto Petrizzo et al., 2019 [21]	Prospective	110	HFS	Knowledge retention	United States
Pawłowicz et al., 2020 [22]	RCT	140	HFS vs. traditional	Student confidence	Poland
Cortegiani et al., 2015 [23]	Prospective	160	HFS vs. LFS	Skill acquisition	Italy
Littlewood et al., 2013 [24]	Prospective	80	HFS	Knowledge retention	United States
Denadai et al., 2014 [25]	RCT	60	HFS vs. LFS	Skill acquisition	Brazil
Choi and Wong, 2019 [26]	Prospective	120	HFS vs. Traditional	Student confidence	South Korea
Heitz et al., 2009 [27]	RCT	50	HFS	Skill acquisition	United States
Gauthier et al., 2019 [28]	RCT	75	HFS vs. LFS	Knowledge retention	France
Nimbalkar et al., 2015 [29]	Prospective	90	HFS vs. traditional	Student confidence	India
Harris et al., 2014 [30]	RCT	100	HFS	Knowledge retention	United States
Alluri et al., 2016 [31]	RCT	200	HFS vs. LFS	Skill acquisition	Brazil
Couto et al., 2015 [32]	Prospective	85	HFS	Student confidence	South Korea
Yu et al., 2021 [33]	Prospective	34	HFS	Student confidence	South Korea
Pal et al., 2024 [34]	RCT	111	HFS vs. video-assisted lecture	Skill acquisition	Malaysia
Offenbacher et al., 2022 [35]	Prospective	50	HFS vs. LFS	Knowledge retention	United States
Saha et al., 2025 [36]	RCT	136	HFS vs. video-assisted teaching	Knowledge retention	Malaysia
Moliterno et al., 2024 [37]	Prospective	33	HFS vs. traditional	Knowledge retention; student confidence	Brazil
Ajab et al., 2022 [38]	Prospective	12	HFS	Student confidence	United Kingdom
Zargaran et al., 2022 [39]	Prospective	50	HFS	Student confidence; knowledge retention	United Kingdom
Bernardi et al., 2019 [40]	Prospective	104	HFS	Skill acquisition	Italy
Coolen et al., 2012 [41]	RCT	43	HFS vs. traditional	Knowledge retention; student confidence; skill acquisition	Netherlands
Meyers et al., 2020 [42]	Prospective	188	HFS vs. traditional	Knowledge retention; student confidence	United States
Morgan et al., 2006 [43]	Prospective	299	HFS	Student confidence; knowledge retention; skill acquisition	Canada
Muniandy et al., 2015 [44]	Prospective	60	HFS	Knowledge retention	Malaysia
Nachiappan et al., 2020 [45]	Prospective	347	HFS	Knowledge retention; skill acquisition; student confidence	Malaysia
Naylor and Torres, 2019 [46]	RCT	152	HFS vs. LFS	Student confidence; skill acquisition	Poland
Scholz et al., 2012 [47]	RCT	63	HFS vs. traditional	Skill acquisition	Germany

Risk of Bias

Risk of bias was evaluated using the Cochrane RoB 2 for RCTs and the ROBINS-I tool for non-randomized studies [48,49]. Among the 47 included studies, 21 were randomized trials and 26 were non-randomized studies. Most RCTs demonstrated low risk of bias in domains related to randomization and outcome reporting; however, blinding of participants and personnel was frequently inadequate, leading to moderate overall risk in several studies. For non-randomized studies, moderate risk of bias was commonly observed due to potential confounding and unclear measurement procedures. A detailed summary of the risk of bias assessment is presented in Table 2.

**Table 2:** Risk of bias assessment of included studies.

Study	Bias due to confounding/randomization (selection bias)	Bias in intervention/deviations (performance bias)	Bias due to missing data	Bias in measurement of the outcome/detection bias
Barsuk et al., 2009 [5]	Low	High	Low	Low
Conlon et al., 2014 [6]	Moderate	Low	Low	Moderate
Norman et al., 2012 [7]	Low	Low	Low	Low
Yang et al., 2019 [8]	Moderate	Low	Low	Moderate
Goolsby et al., 2014 [9]	Moderate	Low	Low	Moderate
Massoth et al., 2019 [10]	Low	Low	Low	Low
Lefor et al., 2020 [4]	Moderate	Low	Low	Moderate
McCoy et al., 2019 [11]	Low	High	Low	Low
Kodikara et al., 2019 [12]	Moderate	Low	Low	Moderate
Feinstein et al., 2001 [13]	Unclear	High	Low	Moderate
Armenia et al., 2018 [14]	Low	High	Low	Low
Adler et al., 2007 [15]	Moderate	Low	Low	Moderate
Price et al., 2010 [16]	Low	Low	Low	Low
Arcoraci et al., 2019 [17]	Low	Low	Low	Low
Holling et al., 2015 [18]	Moderate	Low	Low	Moderate
Nicolaides et al., 2020 [19]	Low	High	Low	Low
Swamy et al., 2014 [20]	Low	Low	Low	Low
Cavuoto Petrizzo et al., 2019 [21]	Moderate	Low	Low	Moderate
Pawłowicz et al., 2020 [22]	Low	High	Low	Low
Cortegiani et al., 2015 [23]	Moderate	Low	Low	Moderate
Littlewood et al., 2013 [24]	Moderate	Low	Low	Moderate
Denadai et al., 2014 [25]	Low	Low	Low	Low
Choi and Wong, 2019 [26]	Moderate	Low	Low	Moderate
Heitz et al., 2009 [27]	Low	High	Low	Low
Gauthier et al., 2019 [28]	Low	Low	Low	Low
Nimbalkar et al., 2015 [29]	Moderate	Low	Low	Moderate
Harris et al., 2014 [30]	Low	Low	Low	Low
Alluri et al., 2016 [31]	Low	Low	Low	Low
Couto et al., 2015 [32]	Moderate	Low	Low	Moderate
Yu et al., 2021 [33]	Moderate	Low	Low	Moderate
Pal et al., 2024 [34]	Low	High	Low	Low
Offenbacher et al., 2022 [35]	Moderate	Low	Low	Moderate
Saha et al., 2025 [36]	Low	High	Low	Low
Moliterno et al., 2024 [37]	Moderate	Low	Low	Moderate
Ajab et al., 2022 [38]	Moderate	Low	Low	Moderate
Zargaran et al., 2022 [39]	Moderate	Low	Low	Moderate
Bernardi et al., 2019 [40]	Moderate	Low	Low	Moderate
Coolen et al., 2012 [41]	Low	Low	Low	Low
Meyers et al., 2020 [42]	Moderate	Low	Low	Moderate
Morgan et al., 2006 [43]	Moderate	Low	Low	Moderate
Muniandy et al., 2015 [44]	Moderate	Low	Low	Moderate
Nachiappan et al., 2020 [45]	Moderate	Low	Low	Moderate
Naylor and Torres, 2019 [46]	Low	High	Low	Low
Scholz et al., 2012 [47]	Low	Low	Low	Low

Skill Acquisition

Skill acquisition is one of the most commonly reported outcomes in studies comparing HFS with LFS. HFS has been shown to improve technical and procedural skills in several high-stakes scenarios, such as central line insertion, airway management, and cardiopulmonary resuscitation (CPR) [5]. For example, Barsuk et al. demonstrated that students who trained with HFS showed significantly higher proficiency in performing central line insertions than those trained using traditional methods or LFS [5].

Similarly, studies focusing on emergency medicine and obstetrics found that HFS enhanced the ability of students to manage complex clinical scenarios such as trauma resuscitation and obstetric emergencies [8,12]. For instance, Kodikara et al. found that HFS improved performance in managing shoulder dystocia, a critical obstetric emergency, compared to LFS and traditional teaching [12].

Despite these benefits, some studies have suggested that LFS may provide similar outcomes for basic procedural skills. For example, Massoth et al. found no significant differences between students trained with HFS and those trained with LFS in terms of basic skills such as intravenous cannulation and suturing [10]. Furthermore, a study compared HFS with video-assisted lecture (VAL) in diagnosing and managing tension pneumothorax [34]. There were no differences in acquiring and retaining the skills in the undergraduate medical students. This raises the question of whether the higher cost of HFS is justified for basic clinical skills training.

Knowledge Retention

The impact of HFS on knowledge retention is less clear. Several studies found no significant differences in knowledge retention between students trained with HFS and those trained with LFS or traditional methods [6,7,35,37]. For example, Conlon et al. conducted a prospective study comparing knowledge retention in students trained with HFS and LFS and found no significant differences after a six-month follow-up [6].

One study compared the use of HFS and video-assisted teaching for ECG lead placement, recording, and subsequent management [36]. Medical students who participated in the video-assisted teaching had better knowledge acquisition compared to those who were trained using HFS.

However, other studies have reported that the immersive nature of HFS may facilitate better integration of theoretical knowledge with practical skills, particularly in high-pressure scenarios [8]. Yang et al. found that HFS improved knowledge retention in students who were trained to manage acute medical emergencies compared to those trained with LFS [8]. Overall, while HFS may offer some advantages in specific contexts, the evidence suggests that its benefits for knowledge retention are not significantly greater than those of LFS for most learning objectives.

Student Confidence

One of the consistently reported benefits of HFS is the increase in student confidence, particularly in managing complex clinical situations. Goolsby et al. found that students who trained with HFS reported higher levels of confidence in their ability to manage critically ill patients compared to those trained with LFS or traditional methods [9]. This is particularly important in fields such as emergency medicine, where confidence is critical for making rapid, life-saving decisions. Yu et al. also reported that medical students were more confident in facing real-time clinical situations in managing patients with pulmonary and gastrointestinal symptoms [33].

However, several studies have raised concerns about the potential for HFS to foster overconfidence in students. Massoth et al. reported that students trained exclusively with HFS were more likely to overestimate their clinical abilities, which could lead to errors in real clinical practice [10]. This suggests that while HFS can boost confidence, it must be complemented by real-world clinical experience to avoid the risk of overconfidence.

Cost-Effectiveness

One of the major challenges associated with HFS is its high cost. Advanced manikins such as the SimMan 3G (Laerdal Medical, Stavanger, Norway) are expensive to purchase, maintain, and operate [9]. Lefor et al. found that LFS could provide comparable educational outcomes for basic skills training at a fraction of the cost, raising questions about the cost-effectiveness of HFS for all stages of medical training [4].

Many medical schools have adopted a tiered approach to simulation training, using LFS for foundational skills and reserving HFS for high-stakes scenarios where the immersive experience and higher fidelity are most beneficial [11]. This approach allows institutions to balance educational outcomes with cost considerations.

During the COVID pandemic, HFS use helped students to improve their confidence level in clinical examination in a time when traditional bedside teaching could not happen as usual [39]. HFS can also be beneficial to train students for scenarios where patients are too unwell or in rare cases [38].

Discussion

This systematic review sought to explore the effectiveness of HFS in UME, focusing on four main areas: skill acquisition, knowledge retention, student confidence, and cost-effectiveness. While the evidence suggests that HFS provide valuable learning experiences, especially in high-stakes clinical environments, the findings also highlight important considerations regarding their optimal use, particularly when compared to LFS and traditional teaching methods.

Skill Acquisition: High Stakes vs. Basic Skills

One of the primary benefits of HFS is its ability to enhance the acquisition of complex technical and procedural skills. As demonstrated in multiple studies, HFS significantly improve student performance in managing critical scenarios such as trauma resuscitation, advanced airway management, and obstetric emergencies [5,8,12]. This is particularly important in medical specialties where real-life exposure to such high-pressure situations may be limited during medical school training, making simulation an invaluable tool for bridging this gap. 

For example, Barsuk et al. showed that HFS-trained students achieved superior outcomes in central line placement, a procedure that carries significant risks if performed incorrectly [5]. Similarly, studies in obstetrics found that HFS improved the management of shoulder dystocia, a rare but dangerous emergency [12]. This supports the idea that HFS can help prepare students for clinical situations that they may rarely encounter during their formal training but must be prepared to handle competently once they begin their careers.

Studies comparing the use of HFS and VAL in undergraduate medical teaching are scarce in the literature. The only one study that did so did not show any advantage of HFS over VAL in skills retention in the management of tension pneumothorax [34]. Repetitive practice of the procedure might consolidate skill acquisition more than the realism provided by HFS.

However, the advantage of HFS over LFS for more basic procedural skills, such as intravenous cannulation and suturing, is less clear. Several studies, including those by Massoth et al., found no significant difference in skill acquisition between students trained with HFS and those using LFS for basic tasks [10]. LFS, which includes simpler models and task trainers, provide sufficient realism for mastering fundamental techniques, which raises questions about whether the higher cost of HFS is justified for these basic educational objectives.

Knowledge Retention: Mixed Results

While the benefits of HFS for skill acquisition are well-documented, their impact on long-term knowledge retention is less consistent. Several studies found no significant difference in knowledge retention between students trained with HFS and those who used LFS or traditional didactic methods [6,7,35]. Traditional methods of teaching were found to be sufficient to build theoretical knowledge [37]. Conlon et al. also reported no meaningful differences in long-term knowledge retention between groups trained using HFS and LFS, suggesting that for cognitive learning, the additional realism offered by HFS may not confer significant advantages [6]. Additionally, one study highlighted the fact that video-assisted teaching might actually enhance knowledge acquisition as undergraduate medical students could access the material as often as they needed, thus allowing them to reinforce their knowledge [36].

On the other hand, certain studies, particularly those involving high-stakes or emergency scenarios, suggest that HFS may enhance knowledge retention when combined with practical, hands-on application. Yang et al. found that students trained with HFS retained more knowledge in emergency scenarios than their peers trained with LFS [8]. This may be because the immersive nature of HFS more effectively engages students, facilitating the integration of theoretical knowledge into practical skills. In high-stakes scenarios, where rapid recall of information is critical, this kind of learning environment may help reinforce not only what to do but also how to apply it under pressure.

The discrepancy between these findings suggests that the context in which HFS is used may determine its impact on knowledge retention. While HFS may not offer superior cognitive learning benefits in routine or low-stakes scenarios, they appear to be particularly beneficial in complex, integrative clinical environments where knowledge and skills must be applied simultaneously and under stress. This suggests that medical educators should carefully consider the context when selecting the appropriate level of fidelity for different learning objectives.

Student Confidence: The Double-Edged Sword

One of the most consistently reported benefits of HFS is its ability to boost student confidence in managing complex clinical situations [9]. Studies such as those by Goolsby et al. indicate that students who train with HFS report feeling more prepared and confident when faced with critical situations, such as trauma or cardiac arrest [9]. Exposure to HFS can also decrease medical students’ anxiety level, thus improving psychological stability [33]. This increased confidence can translate into better performance in real clinical settings, where hesitation or uncertainty can have serious consequences.

On the other hand, several studies have also warned of the potential for overconfidence, particularly when students are trained exclusively with HFS. Massoth et al. highlighted that while HFS boosts confidence, it may also lead to an inflated sense of competence, especially in students who have had limited exposure to real-life clinical environments [10]. This overconfidence could be detrimental in practice, leading students to overestimate their abilities and make critical errors.

Balancing the benefits of increased confidence with the risks of overconfidence is a key challenge for medical educators. While HFS provide an ideal environment for building clinical skills without the fear of harming patients, they should not replace real-world experience. Simulation-based training must be complemented by clinical rotations and hands-on patient care to ensure that students can accurately assess their skills and limitations. Regular debriefing and reflective learning sessions are crucial components of simulation training, allowing students to critically evaluate their performance and identify areas for improvement.

Cost-Effectiveness: A Critical Consideration

The high cost of HFS is one of the most significant barriers to its widespread adoption in medical schools, particularly in low-resource settings [4]. Advanced HFS, such as the SimMan 3G, can cost tens of thousands of dollars, and the ongoing costs of maintenance, faculty training, and facility requirements further add to the financial burden [11]. Given the growing emphasis on cost-effective medical education, many institutions are questioning whether the benefits of HFS justify their high cost, particularly when LFS can provide similar outcomes for many educational objectives.

Studies such as those by Lefor et al. and McCoy et al. suggest that LFS can deliver comparable educational outcomes for basic skills training at a fraction of the cost [4,11]. This has led to the adoption of a tiered simulation model in many medical schools, where LFS is used for foundational skills and HFS is reserved for more advanced, high-stakes scenarios. This approach allows institutions to balance the educational benefits of simulation with the financial constraints they face.

The question of cost-effectiveness is particularly important in resource-limited settings, where investing in HFS may not be feasible. In these contexts, LFS and other lower-cost simulation methods (e.g., standardized patients, virtual simulations, and video-assisted teaching) can provide effective alternatives, allowing students to develop clinical skills without placing undue strain on institutional budgets.

However, HFS could be the best teaching method in unsafe scenarios, such as a pandemic or a road traffic accident. This will allow students to gain exposure to such patients while being in a safe environment. Using HFS to allow students to have hands-on exposure to rare cases might be another worthwhile example where the benefits of HFS can justify its use.

Future research should focus on the long-term cost-effectiveness of HFS by assessing their impact on clinical performance and patient outcomes. If HFS can demonstrably improve patient care and reduce medical errors in the long run, their initial high cost may be justified. However, such studies are still limited, and more evidence is needed to fully understand the return on investment that HFS provide.

Recommendations for Future Research

While this review provides valuable insights into the benefits and limitations of HFS in UME, several gaps in the current literature remain. First, more research is needed on the long-term clinical outcomes associated with HFS training. While short-term improvements in skill acquisition and confidence have been well documented, it is unclear whether these translate into better clinical performance and patient care in the long run. Longitudinal studies that follow students from medical school into residency and clinical practice would provide important data on the lasting impact of HFS.

Second, future studies should explore the optimal combination of simulation fidelity and real-world clinical exposure. While HFS provide benefits for certain high-stakes scenarios, it is important to determine the most effective balance between simulation-based training and hands-on clinical experience. Understanding how to integrate these two components of medical education will help educators design curricula that maximize both skill acquisition and clinical competence.

Finally, cost-effectiveness studies should continue to be a priority, particularly in low-resource settings. Innovative approaches, such as the use of virtual simulation or hybrid models that combine LFS with elements of HFS, could offer cost-effective alternatives without sacrificing educational quality.

## Conclusions

In conclusion, HFS offer significant benefits for skill acquisition, particularly in high-stakes, complex clinical scenarios where hands-on experience is limited. They also boost student confidence and provide an immersive learning environment that helps students integrate theoretical knowledge with practical skills. However, their high cost and the potential for fostering overconfidence must be carefully managed. LFS provide an effective, cost-efficient alternative for basic skills training, and a tiered approach that combines both HFS and LFS may provide the most effective educational outcomes. Future research should focus on the long-term clinical impact of HFS and explore strategies to optimize the cost-effectiveness of simulation training in medical education.
